# The road to linking genomics and proteomics of pathogenic bacteria: from binary protein complexes to interaction pathways

**DOI:** 10.1186/1471-2105-16-S2-A9

**Published:** 2015-01-28

**Authors:** Sarah L Keasey, Mohan Natesan, Christine Pugh, Teddy Kamata, Stefan Wuchty, Robert G Ulrich

**Affiliations:** 1 Molecular and Translational Sciences Division, U.S. Army Medical Research Institute of Infectious Diseases, Frederick, MD 21702, USA; 2 National Center for Biotechnology Information, National Institutes of Health, Bethesda, MD 20892, USA

## Background

The availability of fully sequenced genomes of many bacterial organisms has enabled mapping networks of binary protein interactions that form the basic building blocks of molecular pathways and dynamic assemblies defining all cellular activities. Few proteome-scale studies have been reported for pathogenic bacteria though, suggesting that a systems-wide network analysis of binary interaction partners could reveal groups of proteins that coordinate to achieve specific biological tasks important to pathogenesis and provide a functional map useful to the discovery of new antibiotics, vaccines, and diagnostic tools.

## Results

We performed a comprehensive proteomics analysis of the pathogenic bacterium *Yersinia pestis *and analytically identified more than 74,000 binary interactions. Using a library of biotinylated recombinant proteins to probe a planar microarray comprised of immobilized proteins that represented approximately 85% (3,552 proteins) of the *Y. pestis *proteome, we measured protein-protein interactions by fluorescence intensity of the laser-scanned microarrays. We obtained kinetic interaction data for >1,600 binary complexes by microarray-based, surface plasmon resonance imaging, and identified several high-affinity (*K_D _*~ nM) interactions. We applied a machine learning algorithm that used previously reported experimental protein-protein interactions from *Escherichia coli *as a training set in order to extract *E. coli*-like interactions from the *Y. pestis *dataset. The node degree distribution of the resulting network, comprised of 2344 interactions between 314 proteins, approximates a power-law distribution typical of scale-free networks. Functional annotation clustering of proteins within the network revealed statistically enriched complexes and pathways involved in diverse biological processes. Among the more notable protein assemblies identified were components of the RNA polymerase enzyme and ribosomes. Small modules of proteins related to various metabolic pathways, as well as previously reported interactions involved in homologous recombination and fatty acid biosynthesis, were also present in the network. Two highly interconnected network sub-regions contained a large percentage of proteins with functions linked to transcription and translation.

## Conclusions

We have systematically identified and analyzed thousands of direct binary protein interactions within *Y. pestis*. This new benchmark data set will serve as a critical tool for the analysis of protein interaction networks functioning within an important human pathogen.

